# Is the German Mittelstand more resistant to crises? Empirical evidence from the Great Recession

**DOI:** 10.1007/s11187-021-00573-7

**Published:** 2021-11-24

**Authors:** Michael Berlemann, Vera Jahn, Robert Lehmann

**Affiliations:** 1 ifo Institute - Leibniz Institute for Economic Research at the University of Munich e.V., Poschingerstr. 5, D-81679 Munich, Germany; 2 Faculty of Economics & Social Sciences, Chair for Political Economy & Empirical Economics, Helmut-Schmidt-University Hamburg, Holstenhofweg 85, D-20039 Hamburg, Germany; 3 Center for Entrepreneurship, Innovation and Transformation, Ruhr-University Bochum, O-Werk, D-44780 Bochum, Germany

**Keywords:** Mittelstand firms, Great Recession, Crisis resistance, E31, G12

## Abstract

In a globalized world with high international factor mobility, crises often spread quickly over large parts of the world. Politicians carry a vital interest in keeping crises as small and short as possible. Against this background we study whether the type of company of owner-managed SMEs, in Germany well-known as Mittelstand firms, helps increasing an economy’s crisis resistance. We study this issue at the example of the Great Recession of the years 2008/2009. Using micro panel data from the ifo Business Survey, we study the comparative performance of Mittelstand enterprises and find supporting evidence for the hypothesis that Mittelstand firms performed more stable throughout the Great Recession than non-Mittelstand firms. We also show that owner-managed SMEs performed significantly better than SMEs and owner-managed large enterprises. Thus, it is rather the combination of firm size and owner-management that leads to more crisis resistance.

## Abstract (plain language summary)

The German Mittelstand is economically more successful compared to SMEs and large enterprises because of its combination of owner-management and firm size. The German Mittelstand is always seen as the “backbone” of the German economy and made responsible for Germany’s ability to cope with large external shocks such as the 2008/2009 global recession. To date, this suggestion is not backed by empirical evidence as common data sources do not incorporate information on the ownership structure of German firms. We enrich the micro panel data from the largest monthly business survey in Germany by two questions on the ownership structure of the interviewed firm and can confirm this suggestion. It turns out that it is not firm size or owner-management alone that leads to a higher crisis resistance but rather the combination of both criteria. Our results have practical and political implications as it is possible with our approach to separate the German Mittelstand from the pool of small- and medium-sized enterprises. The information on the ownership structure in our data can initiate future research activities and shed more light on the relevance of owner-management for the economic success of firms.

## Introduction

The world has seen many crises over the last two decades, among them several global crises such as the burst of the Dot.Com Bubble early this century, the 2008 Global Financial Crisis and, still ongoing when this paper was written, the economic crisis in consequence of the COVID-19 pandemic. Throughout these crises, economies typically experience significant drops in their gross domestic products and quickly rising unemployment rates. While in general drops in economic activity are almost inevitable in crisis situations, policymakers around the globe have a vital interest to keep the resulting recessions as small and short-lived as possible. It is thus an intriguing question, how a high degree of crisis resistance can be achieved.

In this paper we study whether the type of company of the German Mittelstand firm can contribute to a higher degree of crisis resistance. It has often been argued that Mittelstand firms are contributing to more stability throughout crises as they are managed by their owners and thus typically have a much longer optimization perspective than manager-led firms, which are often more interested in short-term profits. The fact that Germany was hit very seriously by the 2008 Global Financial Crisis (primarily due to the export orientation of the German economy) but recovered quickly from the crisis and emerged stronger than before indicates that this explanation might be true (see, e.g., Blackstone & Fuhrmans 2011; Girotra & Netessine, 2013). Similarly, in their detailed analysis of the reasons behind Germany’s remarkable performance in the aftermath of the 2008 crisis, Audretsch and Lehmann (2016) dignify the role of the Mittelstand firm as one of the “seven secrets of Germany”. However, several other explanations have been proposed such as German price competitiveness due to wage suppression (Lapavitsas et al., 2011; Bibow, 2013), technological competitiveness (Storm & Naastepad, 2015) and the existence and usage of flexible labor market instruments such as short-time work (Reisenbichler & Morgan, 2013).

Interestingly enough, the hypothesis that Mittelstand firms are more crisis-resistant than other types of firms has never been proved empirically. The major reason why there is comparatively little empirical research on Mittelstand firms in general is that Mittelstand firms, defined as owner-managed small- and medium-sized enterprises (SMEs),^1^ are hard to identify in most available datasets (Berlemann et al., 2018).^2^ As a consequence, most existing empirical studies focus solely on firm size which is much easier to observe. However, this comes at the price that other important characteristics such as the ownership and management structure are neglected.

This paper aims at filling this gap in the literature by analyzing the crisis resistance of Mittelstand firms versus non-Mittelstand firms, thereby taking firm size as well as ownership and management structure into account. We deliver empirical evidence on the performance of German Mittelstand firms throughout the Great Recession in 2008/2009. We base our empirical study on a panel of firm data from the monthly conducted ifo Business Survey, which incorporates a representative sample of 9,000 firms, located all over Germany. By adding a number of special questions to the standard questionnaire we are able to identify Mittelstand firms adequately. Using a panel ordered logit model with interaction effects we find that (on average) Mittelstand firms report a less deteriorating business situation than non-Mittelstand firms over the crisis period. We also show that owner-managed SMEs performed significantly better than SMEs and owner-managed large enterprises. Thus, firm size and owner-management in combination lead to more crisis resistance.

The paper is organized as follows. In Section 2 we explain the concept of the Mittelstand firm in more depth. Section 3 delivers a review of the related literature. After introducing the employed dataset in Section 4, we turn to the empirical analysis in Section 5. In Section 6 we present a number of robustness tests. Section 7 summarizes the main results and concludes.

## The German Mittelstand firm

As there is much confusion on the exact meaning of the term “Mittelstand” we explain it in detail before we turn to our subsequent empirical analysis. Up to now, there is no legal or otherwise generally accepted definition of Mittelstand firms (Becker & Ulrich, 2011; Pahnke & Welter, 2019). However, there is a lively and quite controversial scientific discussion on the question how Mittelstand companies can be defined adequately. Some authors (e.g., Hausch 2004; Pfohl 2006; Damken 2007) suggest extensive lists of criteria for the identification of Mittelstand enterprises. Pfohl (2006), for example, elaborates the qualitative characteristics of SMEs on the basis of the criteria of procurement, production management, marketing, innovation management, human resources management, logistics, financing, and controlling. However, these lists are typically rather descriptive than delivering theoretical arguments why Mittelstand firms are a superior form of organizing certain businesses. Moreover, extensive criteria lists have little practical use as the necessary data requirements to distinguish Mittelstand from non-Mittelstand firms are extraordinarily high. Other studies (e.g., Wolter and Hauser 2001; Icks 2006; Becker & Ulrich 2011) focus on fewer, particularly central features. As an example, Icks (2006) names the unity of the economic existence of the company and its management as well as the responsible participation of the management in all decisions relevant to corporate policy as central qualitative criteria. Indeed, the unity of ownership and management of companies plays a significant or even central role in all definitions.

Against this background one might ask why owner-management is seen as constitutive element of Mittelstand firms. Owner-managed companies have, on the one hand, the advantage that the managing owner will direct his actions completely towards the company’s success (Alchian and Demsetz, 1972). The owner-manager bears all the consequences of his management decisions and thus has a strong incentive to make the best possible decisions for the company. If, on the other hand, the owner of a company instructs a manager to run his company, a principal-agent relationship evolves between owner and management (Jensen & Meckling, 1976). While the owner is often interested in the long-term maximization of the company’s value, the manager usually has a shorter optimization perspective. Since the manager is deeply involved in operational business, he has a considerable information advantage over the owner, which gives him the opportunity to pursue own goals. In order to ensure that the manager acts in the interests of the owner, control measures must be applied. Depending on the degree of the existing information asymmetry between owner and manager, the costs of these measures can be considerable. As a rule, perfect control is neither possible nor economically viable. Ultimately, the great advantage of the unity of ownership and management lies in the avoidance of principal-agent problems between owners and managers of companies.

While, for example, the *Institut fuer Mittelstandsforschung Bonn* (IfM) refers solely to the unity of ownership and management in its definition of the Mittelstand, other definitions also include firm size (Berlemann et al., 2007; Becker & Ulrich, 2011; Berlemann & Jahn, 2016; Jahn, 2018). This is due to the argument that Mittelstand firms can only show their strengths in terms of high flexibility and short decision-making paths if the company does not exceed a certain size, as size is connected to various forms of internal company transaction costs (Ewers et al., 2003). Organizational costs can play an important role here. While there will be hardly any organizational costs with a small production volume and highly standardized products, this is usually different as the company grows. Additional hierarchy levels are often necessary in order to delegate decision-making power and to organize production and sales. The individual organizational units have to coordinate and agree and thereby generate transaction costs (e.g., the working time spent in meetings). An increase in the size of the company is often accompanied by a decline in the manageability of the company (information asymmetries) and the resulting control errors. With the growing size of a company, the processes become more complex, the amount of information to be evaluated larger and the information paths longer. This increases the coordination effort and causes a company to react less quickly (Schachner et al., 2006). There is also often a significant internal bureaucracy that is typically associated with internal inefficiency. Employee motivation can also suffer if the employee’s individual contribution to the company’s overall output becomes increasingly smaller. Against the background of this argument, it seems to be reasonable to exclude large companies from the group of Mittelstand firms. In our subsequent empirical analysis, we follow this approach and define Mittelstand firms as owner-managed small- and medium-sized enterprises (see Section 4.2 for the detailed identification procedure).

## Related literature

To the best of our knowledge, up to now there are no quantitative studies analyzing the resilience of Mittelstand firms in economic downswings. We are the first to estimate firm performance of Mittelstand firms, defined as owner-managed SMEs, in comparison to non-Mittelstand firms in recessions. Consequently, there is no empirical study that is closely related to ours and could be presented in this literature review. However, some empirical analyses are connected to ours since they study the two features of Mittelstand firms, owner-management and a small firm size, separately. The first strand of the literature focuses on the effect of ownership structure on firm performance in economic downturns whereas the second analyzes the relationship between firm size and firm performance in recessions.

Table 1 presents an overview of these two literature strands and connects their results to our empirical analysis. The upper part of the table focuses on studies about ownership structure and firm performance in recessions. This strand of the literature is small and the results quite heterogeneous. Studies especially differ in the way they measure firms’ ownership structures. None of the existing studies includes all owner-managed firms. Some studies analyze the performance of so-called “founder firms”, i.e., the subgroup of owner-managed firms where the firm’s founder is managing the firm’s business (see Bartz & Winkler 2016; Zhou et al.^2017^).^3^ Other studies focus on family firms (see Cowling et al., 2015; Minichilli et al., 2016). While many family firms are factually owner-managed, this does not hold true for all family firms. In some family firms parts of the family own at least parts of the company while other family members manage it. Thus, ownership and management are not necessarily combined in the same person.^4^ Moreover, there are numerous firms which are owner-managed but not family firms. In order to structure this heterogeneous literature, Table 1 presents the respective papers according to the measurement of firms’ ownership structure used (see column 2, highlighted in italics). Column 2 reports the main results of a particular study. Column 3 briefly summarizes the arguments, the authors provide to explain their empirical findings. The last column links the respective analysis to ours. It reports the sign we should find for Mittelstand firms, based on the study’s findings.

**Table 1:** Overview of related literature and link to our analysis

Article	Main results	Explanation	Hypothesis
LITERATURE ON OWNERSHIP STRUCTURE			
Bartz and Winkler (2016)	*Founder firms* experience a stronger decline in growth		(-)
Zhou et al. (2017)	*Founder firms* outperform *non-family firms*	Founder firms are more risk averse, do not have to bear agency costs, and have better access to the capital market	(+)
Cowling et al. (2015)	*Family ownership* does not have a significant effect		(n.s.)
Minichilli et al. (2016)	*Family firms* outperform non-family firms	Family firms are long-term oriented, have tacit knowledge about the firm’s identity and close relationships to customers, suppliers, employees, and banks	(+)
Cesaroni et al. (2017)	*Family firms* perform worse than non-family firms	Family firms have only restricted managerial skills and try to ensure workplaces for family members	(-)
Hansen et al. (2020)	No significant difference between *family firm* performance and non-family firm performance in Continental Europe and Japan		(n.s.)
LITERATURE ON FIRM SIZE			
Gertler and Gilchrist (1994)	Especially small firms experience sharp declines in sales	Small firms suffer from liquidity constraints	(-)
Fort et al. (2013)	Young, small businesses experience larger declines in employment	Young, small firms have a more local customer base and face stronger credit constraints	(-)
Cowling et al. (2015)	Especially larger SMEs with sound access to finance manage to grow		(-)
Peric and Vitezic (2016)	Significantly positive relation between firm size and turnover growth		(-)
Varum and Rocha (2011)	Significantly negative relation between firm size and employment growth	Large firms are the first to lay-off workers	(+)
Varum and Rocha (2013)	Larger firms are more heavily affected by recessions		(+)
Bartz and Winkler (2016)	Relative growth advantage for small firms	Small firms are more flexible	(+)

*Notes:* The second column presents the main results on firm performance during economic crises. The third column briefly summarizes the arguments the authors provide to explain their empirical findings. The last column shows the conclusion we draw from the respective study for our own empirical analysis. Based on the related literature, we expect Mittelstand firms to perform significantly worse (-), significantly better (+), or similar to (n.s.) other types of businesses in a recession

Zhou et al. (2017), for example, find founder firms to show higher operating returns on assets than non-family firms during a recession. The authors argue this finding to be due to a higher degree of risk aversion of founder firms, which leads to less investments during a crisis. They argue that conflicts of interests between long-term oriented firm owners and myopic managers in non-family firms are highly costly in economic downturns. Managers would be likely to boost short-term earnings through over-investment when sales fall during a crisis. This would be extremely risky when the firms rely on bank loans. As banks impose strict lending policies in times of crises, short-term loans might dry up and put ongoing projects under pressure. Moreover, Zhou et al. (2017) explain the outperformance of founder firms in economic downturns through a better access to the capital market during a crisis. Established relationships between long-term oriented founder firms and financial institutions might help founder firms to get money even in times of economic downturns. They would thus be able to invest in promising projects even in economically hard times. Given the empirical findings of Zhou et al. (2017), we should expect owner-managed firms to outperform non-owner-managed businesses in economic downturns. Since owner-management is one central criterion of Mittelstand firms, we suppose Mittelstand firms to outperform non-Mittelstand firms in economic crises as well, which we indicate with a (+) in Table 1, column 4. Cesaroni et al. (2017), as another example, find that non-family firms in Italy tend to perform better than family firms in the Great Recession 2009. They provide two reasons for this finding. First, managerial skills in family firms would be restricted to those possessed by family members. However, especially in economic crises these skills would be crucial. Second, even in a recession family firms would try to ensure workplaces for family members in order to cultivate relationships within the family. The results of Cesaroni et al. (2017) indicate that Mittelstand firms perform worse in the Great Recession than non-Mittelstand firms, which is denoted with a (-) in Table 1, column 4. These two examples concretely demonstrate that the literature on ownership structure and firm performance in recessions is heterogeneous in its results, and consequently leads to different assumptions about the performance of Mittelstand firms compared to non-Mittelstand firms in economic crises.

The lower part of Table 1 addresses the empirical literature on the relation between firm size and performance in economic downturns. Just as the literature on ownership structure, the results of this literature strand are again mixed. Some studies find a significantly positive relationship between firm size and performance during economic crises (see Gertler and Gilchrist 1994; Fort et al., 2013; Cowling et al., 2015; Peric & Vitezic^2016^). Fort et al. (2013), for example, study net employment growth of U.S. firms of different sizes and ages throughout the 2007–2009 recession. Their analysis finds that especially young, small businesses experience large declines in employment during the crisis and thus seem to be more vulnerable to economic shocks than their large, mature peers. As possible mechanisms behind this result the authors consider a customer base that is more local and stronger credit constraints for small and young businesses. Small and young firms are likely to produce goods and services for a limited geographic area (e.g., a small restaurant) and are thus especially prone to local cyclical shocks. Other studies reveal a significantly negative relation between firm size and firm performance in economic downturns (see Varum and Rocha 2011; Varum & Rocha 2013; Bartz & Winkler 2016). Bartz and Winkler (2016), for example, analyze turnover and employment growth of small- and medium-sized firms in Germany in the crisis year 2009 relative to a period of economic stability in 2006. They discover a relative growth advantage for small firms compared to larger businesses in both stable and crisis times. However, the economic crisis reinforces the relative growth advantage. Bartz and Winkler (2016) explain this result with a higher flexibility of small firms, which is especially valuable in crisis times. The empirical literature on firm size and firm performance in recessions is thus heterogeneous in its results. Since a small firm size is one central feature of Mittelstand firms, we conclude that the relation between Mittelstand firms and performance in economic crises is an open question as well.

In summary, empirical results of both strands of the literature tend to be mixed. On the one hand, owner-management can positively influence firm performance in economic downturns since risk averse and long-term oriented owners tend to make more careful investment decisions and often have better access to the capital market in recessions than manager-led firms. Due to established relations to long-term oriented owners, financial institutions might be willing to provide money even in economically hard times (Zhou et al., 2017). On the other hand, owner-managed firms might have limited human resources because managerial skills are restricted to those possessed by family members. Managerial skills, however, are essential in economic downturns (Cesaroni et al., 2017). Moreover, small firms have advantages as well as disadvantages in economic crises. While SMEs can benefit from being highly flexible (Bartz & Winkler, 2016), they might suffer from a more local customer base and stronger credit constraints compared to larger businesses (Fort et al., 2013; Smallbone et al., 2012; Cowling et al., 2015; Gertler & Gilchrist, 1994). Thus, it remains an open question whether owner-managed SMEs, respectively Mittelstand firms, perform significantly better, worse or similar to other types of businesses in economic crises.

## Data

### The ifo Business Survey

We base our analysis on the monthly business survey conducted by the German ifo Institute.^5^ Each month, the ifo Institute surveys a relative stable sample incorporating 9,000 answers of German firms, which ensures the survey to be representative for the German economy in terms of firm size and sectoral coverage. Currently, the ifo Business Survey approximately represents 74% of total gross value added (GVA) in 2018, with business cycle indicators for the four main sectors manufacturing, construction, trade, and services (see Lehmann^2020^).^6^

The ifo Institute targets its survey on the product level instead of the firm level. Whenever a firm supplies only one product, both concepts coincide. If a firm, however, has multiple product lines or variations of one product category, the ifo Institute surveys this firm multiple times and for each product separately. This is a crucial differentiation with which we have to deal when it comes to identifying Mittelstand firms. Appendix Appendix contains the details on the data set preparation.

Generally, the ifo questionnaire is divided into standard questions, which are comparable across the products, and special questions.^7^ The standard questions are asked regularly, i.e., either each month, quarterly, bi-annually or annually. Special questions are added only occasionally, often as a part of some sort of special investigation of a certain topic. As a general rule, questions asked by the ifo Institute are of *qualitative* nature.

Our subsequent empirical analysis focuses on the current business situation. The wording of the assessment of the current business situation (*ifo Business Situation*) for each product of the firms is as follows: “We assess our current business situation as [...]”. Each respondent can choose from three different, qualitative answers reflecting either a positive, neutral, or negative assessment. The three answers for the business situation are as follows: (+) good, (=) satisfactory, and (–) bad. We decided to choose the business situation as an indicator of firm performance mainly because of the findings from the forecasting literature. The survey on the forecasting performance of the ifo data by Lehmann (2020) reveals that the ifo Business Situation has high predictive power for the development of economic indicators (for example, sales or gross value added) across industries and subgroups, thus, it serves as a measure of economic activity. Further studies, and only to name a few, attest survey data to have high predictive power for economic aggregates (Angelini et al., 2011; Basselier et al., 2018) or to track economic activity (see, for example, de Bondt 2019). The ifo Institute also ran a special survey among their trading firms to investigate on which indicators the firms build their answer for the business situation (see Abberger et al., 2011). The survey revealed that the firms build their assessment either on current sales or on their current profit situation. Based on the large forecasting literature and the special survey results, the ifo Business Situation is a suitable indicator to measure economic activity across firm types.

Moreover, the ifo Business Survey has several advantages that make it suitable for our research question. First, in order to investigate the resistance of firms in economic recessions, we need timely disaggregated information and thus variation over the business cycle. As the survey is conducted each month, we can accurately identify the crisis period of the years 2008/2009. As profits etc. are typically measured on an annual basis, this information does not help us to identify crisis effects as business cycle fluctuations are also within-year phenomena. Second, profit data only have to be reported by a rather small number of firms in Germany due to their obligation to provide information. Thus, annual profit information is not available for most German firms, especially smaller ones. The ifo Business Situation, however, is comparable across firms and products, allowing us to analyze a measure of economic performance for many firms of different sizes. And third, we are able to ask special questions that allow us to identify the ownership structure of the firm, which is an information typically unavailable in official statistics. The identification of the Mittelstand via the survey is described in the next section. In the end, the ifo Business Survey consistently allows us to investigate the economic performance of many firms in different industries, of different sizes and each month.

### Identification of Mittelstand firms

For our empirical analysis it is necessary to distinguish between Mittelstand and non-Mittelstand firms. As explained earlier, we follow the idea to define Mittelstand firms as owner-managed small- and medium-sized enterprises (Berlemann et al., 2007; Becker and Ulrich, 2011; Berlemann & Jahn, 2016; Jahn, 2018). More precisely, we base our analysis on the definition proposed by Berlemann et al. (2007) and classify a firm as belonging to the Mittelstand whenever the following three criteria are met simultaneously:

1. the firm has less than 500 employees,
2. the firm has a maximum of four managers, and
3. at least one of maximal four managers owns company shares.

The first criterion focuses on firm size and aims at identifying SMEs. According to the definition of the *Institut fuer Mittelstandsforschung Bonn* firms are classified as SME whenever they have less than 500 employees and realize a turnover less then 50 million €. However, as the ifo Business Survey does not cover any information on turnover figures, we exclusively use employment figures to classify firms as SME or as large enterprise. According to the findings reported in Berlemann et al. (2018) this procedure should be unproblematic as the authors find almost the same SME-quotas when exclusively using employment figures or by applying both criteria, employment and turnover. Thus, the inaccuracy we have to accept by exclusively using the employment criterion when identifying SMEs should be negligible. We, however, come back to this issue in the section covering the robustness checks.

The second and the third criterion focus on the internal structure of the firm, and here especially on whether it is owner-managed or not. As explained earlier, the major advantage of Mittelstand firms is the unity of ownership and management, which firstly prevents principal-agent problems from occurring. Secondly, the lean organization of owner-managed fims keeps transaction costs low and allows quick reactions to changing market conditions. Many firms have a single owner-manager and thus are obviously fulfilling the criterium of owner-management. However, firms can also have various owner-managers. As an example, this holds true in many family firms where often various family members own a firm and at the same time are also engaged in its management. However, firms might also be owned and managed by individuals without any family ties. Often firms are founded by individuals with complementing abilities. As long as the number of owner-managers is comparatively low, there is little reason to believe that the advantages of owner-management are lost by having a few owners which are engaged in a firm’s management. Quite the contrary, having more than one owner-manager can often be beneficial in the case of absence of an owner-manager (e.g., illness, business travels, holidays). However, the transaction costs within the firm tend to increase with a larger number of involved chief operating officers. We therefore follow the related empirical literature and restrict the maximum number of owner-managers allowed to be classified as a Mittelstand firm to four (see, e.g., Berlemann et al.^2007^).

As the ifo Business Survey itself contains no information on the ownership structure of the surveyed firms but offered us the possibility to ask special questions as stated in the previous section, we collected the necessary information on owner-management through two questions with the wording:

1. “Is your enterprise managed by more than four people?”
2. “Owns at least one of the manager company shares?”

For the manufacturing and the wholesale and retail trade sector, the special questions were included in August 2016. Firms from the construction and services sector were asked in September 2016. In total, 5,845 firms answered the special questions.

**Fig. 1:**
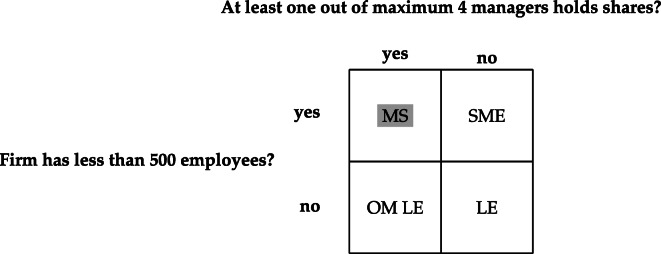
Firm types. LE, large enterprise; MS, Mittelstand; OM, owner-managed; SME, small- and medium-sized enterprise

Based on the number of employees and the answers to the questions on owner-management, firms can be classified as either Mittelstand firms (MS), non-owner-managed SMEs (SME), owner-managed large enterprises (OM LE) or as non-owner-managed large enterprises (LE) (see Fig. 1).

The results of the classification of firms is shown in Table 2. Based on the earlier described criteria, 64.5 percent of all firms were classified as Mittelstand firms. From the remaining 2,076 enterprises, 67.2 percent can be classified as non-owner-managed SMEs (SME), 8.2 percent are owner-managed large enterprises (OM LE), and 24.6 percent count as non-owner-managed large enterprises (LE).

**Table 2:** Dataset by firm types

Firm type	Number of firms	Share (in %)
Mittelstand firms (MS)	3,769	64.5
Non-Mittelstand firms	2,076	35.5
*Among them:*		
Non-owner-managed SMEs (SME)	1,395	67.2
Owner-managed large enterprises (OM LE)	170	8.2
Non-owner-managed large enterprises (LE)	511	24.6
All firms	5,845	100.0

## Estimation strategy and results

### Empirical approach

Our aim is to study whether Mittelstand firms performed significantly different from non-Mittelstand firms throughout the Great Recession. Our baseline estimation approach therefore consists of estimating the following interaction model,

$$ \begin{array}{@{}rcl@{}} \text{Performance}_{it} & = & a_{i} + \delta_{1} \cdot \text{MS}_{it} + \delta_{2} \cdot \text{Crisis}_{t} \\ &&+ \delta_{3} \cdot \text{MS}_{it} \cdot \text{Crisis}_{t} \\ & & + \sum\limits_{j=1}^{J-1}{\beta_{j} \cdot \text{State}_{ij}}\\ && + \sum\limits_{k=1}^{K-1}{\gamma_{k} \cdot \text{Sector}_{ik}} + \varepsilon_{it} {,} \end{array} $$

where Performance_*it*_ is the measure for economic performance of firm *i* at time *t*. MS_*it*_ is a Mittelstand dummy which takes the value of one when firm *i* belongs to the German Mittelstand (and zero otherwise).^8^ The dummy Crisis_*t*_ controls for the worldwide financial crisis period and takes the value of one throughout the crisis period (and zero otherwise). According to the German Council of Economic Experts (*Sachverstaendigenrat zur Begutachtung der gesamtwirtschaftlichen Entwicklung*), the crisis period lasted from April 2008 to March 2009. We coded the crisis dummy accordingly. State_*ij*_ controls for state-specific effects by a set of *J* − 1 dummy variables indicating whether firm *i* is located in state *j*. Similarly, Sector_*ik*_ controls for sector-specific effects. *ε*_*it*_ represents the usual idiosyncratic error term and *a*_*i*_ the unobserved heterogeneity at the firm level. Our period under investigation covers January 2006 to March 2009.

As firm performance measure we employ firms’ reported business situation. The reasoning behind this choice is the following. As stated before, the ifo Business Survey distinguishes between four time dimensions: past, present, three-month and six-month expectations. We decided against the past and future dimensions and apply the present category and thus the business situation as it reflects the current state of the firms’ business performance, given recent developments within a firm. We also did so as the ifo Business Situation has been proven to be a good predictor for a variety of macroeconomic aggregates, for example, sectoral gross value added (see Lehmann 2020, for a literature survey). The wording of questions on the past development does usually imply a change in a specific variable, thus, it proxies the first derivation of the current firm performance and depends on the previous month’s state. The questions regarding future developments also imply a path dependency due to their wording and might only reflect the firms’ ability to rationally assess its future performance, given its characteristics and internal information. If, however, general firm conditions change within the expectation period, it should no longer be a predictor for the firms’ current performance. Therefore, our empirical strategy focuses on current developments within the firms. As explained earlier, the business situation can only take three values that we re-coded in advance to achieve the following order and thus a natural interpretation of the coefficients: “bad”, “satisfactory” and “good”. As a consequence to the variable’s characteristics, we fit an ordered logit model to the data where the unobservable and time-invariant firm characteristics *a*_*i*_ are treated as random effects. Besides owner-management and a small firm size as the core characteristics of Mittelstand firms, the firm’s location and the sector it is operating in, there can be further unobservable business attributes that might have an effect on firm performance and thus drive our overall results.^9^

The coefficients of interest to be estimated are the difference in average performance between Mittelstand and non-Mittelstand firms, *δ*_1_, the effect of the crisis on the average firm performance, *δ*_2_, and the interaction effect of the Mittelstand dummy and the crisis dummy, *δ*_3_. The latter coefficient measures to what extent the performance of Mittelstand firms is affected by the economic crisis in comparison to non-Mittelstand firms. Given that the hypothesis “Mittelstand firms perform better throughout economic crises” is correct, we should find a significantly positive value for *δ*_3_.

### Estimation results

The estimation results are summarized in Table 3.^10^ In the first column we report the estimation results for a model that does not account for state- and sector-specific effects. We find that Mittelstand firms (on average) report a significantly worse business situation than non-Mittelstand firms. Moreover and unsurprisingly, the reported business situation deteriorated throughout the crisis period for the average sample firm. The coefficient of our variable of central interest, the interaction effect, turns out to be positive and highly significant. Thus, the negative effect of the crisis on the business situation of Mittelstand firms turns out to be less severe than for non-Mittelstand firms. In other words, the business situation of non-Mittelstand firms deteriorated much stronger in the Great Recession 2008/2009 than those of the Mittelstand firms. When estimating the model with state- and sector-specific effects the general difference between Mittelstand and non-Mittelstand firms becomes insignificant. Most likely this is due to the fact that the share of Mittelstand firms differs significantly across different sectors. Thus, when controlling for sector-specific effects the average business situation of a Mittelstand firm does not differ from the current performance of a non-Mittelstand firm. However, the effect of the crisis remains almost unchanged. The same holds true for the interaction effect. Thus, our central result carries over to the case where we estimate the model with state- and sector-specific effects.

**Table 3:** Effect of financial crisis on Mittelstand and non-Mittelstand firms

	Dependent variable: business situation	
Model	excl. specific effects	incl. specific effects
Mittelstand firms (*MS*)	− 0.53^∗∗∗^	− 0.14
	(0.10)	(0.10)
Crisis period (*Crisis*)	− 1.32^∗∗∗^	− 1.31^∗∗∗^
	(0.06)	(0.06)
Interaction effect (*MS* ∗ *Crisis*)	0.43^∗∗∗^	0.43^∗∗∗^
	(0.08)	(0.08)
State-specific effects	NO	YES
Sector-specific effects	NO	YES
Pseudo R-squared	0.22	0.19
Observations	85,544	85,502

*Notes:* Ordered logit estimation with robust standard errors in parentheses. ^∗∗∗^, ^∗∗^, and ^∗^ denote statistical significance to the 1%, 5%, and 10% level. The reference is the group of non-Mittelstand firms

So far, we compared the crisis-performance of Mittelstand firms to the group of non-Mittelstand firms. However, the group of non-Mittelstand firms consists of various quite diverse subgroups. In order to find out whether Mittelstand firms systematically differ from non-owner-managed SMEs and owner-managed large enterprises we re-estimate the model taking the four different firm types in our sample (see Table 2) explicitly into account: Mittelstand firms (MS_*it*_), non-owner-managed SME (SME_*it*_), owner-managed large enterprises (OM LE_*it*_), and non-owner-managed large enterprises (LE_*it*_). Different from our baseline regression we use the group of Mittelstand firms as comparison group and study via three separate interaction terms whether the other three groups performed significantly different throughout the crisis.

The referring estimation results are shown in Table 4. In the first column of the table we again show the results for the model without state- and sector-specific effects while the second column includes both types of time-invariant effects. Qualitatively both models deliver very similar results. All three types of non-Mittelstand firms on average report a better business situation than Mittelstand firms. Moreover, the general effect of the crisis turns out to be significantly negative. However, most interesting, all three types of non-Mittelstand firms performed systematically worse throughout the crisis as compared to Mittelstand firms. Thus, our empirical evidence points into the direction that it is the combination of firm size and owner-management which leads to a high degree of crisis resistance, and not firm size or owner-management alone.

**Table 4:** Effect of financial crisis across firm types

	Dependent variable: business situation	
Model	excl. specific effects	incl. specific effects
*SME*	0.42^∗∗∗^	0.16^∗^
	(0.10)	(0.10)
*OM* *LE*	0.56^∗∗∗^	0.32^∗∗^
	(0.14)	(0.14)
*LE*	0.68^∗∗∗^	0.23^∗^
	(0.13)	(0.13)
*Crisis*	− 0.89^∗∗∗^	− 0.88^∗∗∗^
	(0.02)	(0.02)
*SME* ∗ *Crisis*	− 0.12^∗^	− 0.12^∗∗∗^
	(0.07)	(0.04)
*OM* *LE* ∗ *Crisis*	− 0.88^∗∗∗^	− 0.88^∗∗∗^
	(0.09)	(0.09)
*LE* ∗ *Crisis*	− 1.00^∗∗∗^	− 1.00^∗∗∗^
	(0.06)	(0.06)
State-specific effects	NO	YES
Sector-specific effects	NO	YES
Pseudo R-squared	0.22	0.19
Observations	71,174	71,132

*Notes:* Ordered logit estimation with robust standard errors in parentheses. ^∗∗∗^, ^∗∗^, and ^∗^ denote statistical significance to the 1%, 5%, and 10% level. The reference is the Mittelstand

### Identification of Mittelstand firms by self-assessment

Up to now we classified Mittelstand firms based on objective criteria. In the following we study an alternative Mittelstand classification, which is based on a self-assessment of the surveyed firms. This self-assessment (*MS*-self) is gained by an additional special question, we asked within the ifo Business Survey. The wording of this question was “Do you classify your enterprise as part of the German Mittelstand?” Interestingly enough, the results of the self-assessment differ strongly from the classification on objective criteria. While 64.5 percent of all surveyed firms were classified as Mittelstand firms according to the objective criteria, some 83.3 percent of all responding enterprises assess themselves that they belong to the German Mittelstand. Interestingly, only 23.0 percent of all firms that do not fulfill the objective criteria of a Mittelstand firm give a correct self-assessment, thus, 77.0 percent see themselves as a Mittelstand firm whereas they are not according to our objective delimitation. Only a minority of firms (13.7 percent) wrongly classify themselves as non-Mittelstand firms whereas they belong to the Mittelstand based on objective criteria. This especially holds true for very small firms with an average of less than 20 employees (see Berlemann et al., 2018). Welter et al. (2015) attribute this to wrong perceptions of the firms about the Mittelstand. Small firms think that they are too small to be a Mittelstand firm.

The referring estimation results with the assessment of the business situation as dependent variable and shown in Table 5 differ strongly from our baseline estimation. Here, the coefficient of the interaction effect turns out to be zero. We attribute this to the fact that the self-assessment of a large share of firms does not coincide with the objective criteria. As mentioned earlier, many German enterprises understand themselves as Mittelstand firms although they formally do not belong to this type of company because they are either too large or are not managed by their owners. Various reasons might explain the wrong self-assessments of firms as Mittelstand firms. As there is no commonly accepted definition of the term “Mittelstand” in science it would be not too surprising that firms have quite differing characteristics of Mittelstand firms in their mind when making their self-assessments. Moreover, the vast majority of firms starts their business history as owner-managed SME. When these firms grow over time or change their management structures they still might understand themselves as Mittelstand firms although they factually already belong to the group of large or non-owner-managed firms.^11^ However, misclassifications might also be driven by the wish to belong to this firm type as the German Mittelstand has an excellent national and international reputation. Anyway, our empirical results show that crisis stability is not systematically correlated with (erroneous) self-assessments of firms but, rather, with fulfilling the objective criteria of being an owner-managed SME.

**Table 5:** Self-assessment as Mittelstand firm

	Dependent variable: business situation	
Model	excl. specific effects	incl. specific effects
*MS*-self	0.44^∗∗∗^	0.54^∗∗∗^
	(0.16)	(0.14)
*Crisis*	− 1.02^∗∗∗^	− 1.02^∗∗∗^
	(0.11)	(0.11)
*MS*-self ∗ *Crisis*	0.00	0.00
	(0.12)	(0.12)
State-specific effects	NO	YES
Sector-specific effects	NO	YES
Pseudo R-Squared	0.22	0.19
Observations	74,527	74,484

*Notes:* Ordered logit estimation with robust standard errors in parentheses. ^∗∗∗^, ^∗∗^, and ^∗^ denote statistical significance to the 1%, 5%, and 10% level. The reference is the group of firms that reported to be not part of the German Mittelstand

## Robustness checks

In order to check the reliability of our main results, we conduct a number of robustness checks. Again, we use the business situation as dependent variable. First, instead of applying the German SME-definition (threshold 500 employees), we apply the European definition, which refers to a threshold of 250 employees. When coding an additional dummy variable accordingly (*MS*-250) and using it in the regression, we receive the results reported in column (1) of Table 6. All results from our baseline regressions are confirmed by this procedure, thus, Mittelstand firms performed significantly better than non-Mittelstand firms throughout the crisis.^12^

**Table 6:** Robustness checks

	(1)	(2)	(3)	(4)	(5)
Dependent variable: business situation					
*MS*-250	− 0.23^∗∗∗^				
	(0.10)				
*MS*-both		− 0.75^∗∗∗^	− 0.89^∗∗∗^	− 0.96^∗∗∗^	− 0.94^∗∗∗^
		(0.15)	(0.18)	(0.19)	(0.20)
*Crisis*	− 1.29^∗∗∗^	− 1.30^∗∗∗^	− 1.33^∗∗∗^	− 1.32^∗∗∗^	− 1.40^∗∗∗^
	(0.05)	(0.05)	(0.08)	(0.08)	(0.09)
Interaction effect	0.43^∗∗∗^	0.53^∗∗∗^	0.55^∗∗∗^	0.51^∗∗∗^	0.60^∗∗∗^
	(0.07)	(0.14)	(0.16)	(0.16)	(0.18)
Founding year			0.00^∗∗∗^		
			(0.00)		
Equity				− 0.00	
				(0.00)	
Legal form					0.00
					(0.00)
State-specific effects	YES	YES	YES	YES	YES
Sector-specific effects	YES	YES	YES	YES	YES
Observations	95,322	57,210	31,110	29,967	26,232

*Notes:* Ordered logit estimation with robust standard errors in parentheses. ^∗∗∗^, ^∗∗^, and ^∗^ denote statistical significance to the 1%, 5%, and 10% level. The reference is the group of non-Mittelstand firms

Second, we tried to further enhance our objective identification of Mittelstand firms in the dataset by employing information on turnover from additional datasets, which can be combined with the ifo Business Survey data. As explained earlier, the ifo Business Survey contains no such information. However, the data center at the ifo Institute provides the possibility to combine the ifo Business Survey data with information from the Amadeus- and the Hoppenstedt-Database. The latter two databases provide balance sheet and income statement data and also contain a variety of firm characteristics such as firms’ turnover, founding years, their legal forms and their amount of equity capital. The German law defines several legal forms of companies. We can distinguish between twelve different forms, for example, stock corporations (*Aktiengesellschaften*), limited liability companies (*GmbHs*), or limited partnerships (*Kommanditgesellschaften*). According to their balance sheets, the firms report—in addition to their assets and liabilities—the amount of their equities (in thousand Euros). Combining the ifo Business Survey data with the Amadeus and the Hoppenstedt data thus delivers additional firm information, however, this comes at the price of a significantly shrinking sample size. This is due to the fact that many of the firms in the ifo sample are not liable to prepare a balance sheet. This holds true especially for numerous small firms. As a consequence, the number of available observations per cross-section drops to 2,411 firms in the merged dataset. At the same time the SME-quota drops from 91.7 percent in the ifo data to only 53.6 percent in the merged data.

In column (2) of Table 6 we show the results we receive for the merged dataset when using both the employee and the turnover criterion to classify Mittelstand firms (*MS*-both). We receive qualitatively the same results as in the baseline regression. Again the coefficient of the interaction effect turns out to be significantly positive, indicating that Mittelstand firms performed significantly better throughout the crisis than non-Mittelstand firms. The results also remain stable when we additionally include a number of control variables on the firm level such as the founding year, the total amount of equity or the firms’ legal form (see columns (3), (4) and (5) in Table 6).

Third, we ran a bunch of further robustness checks: (i) we included an interaction effect across the state- and sector-specific effects,^13^ (ii) we estimated on the product, instead of the firm, level and applied clustered standard errors, and (iii) we augmented the baseline model by including the lagged value of the firms’ business situation. As for the previous two robustness checks, our main results remain robust to these alternative specifications. All these additional results can be found in Appendix Appendix.

## Conclusions

A remarkable specialty of the German economy is the comparatively large share of owner-managed small- and medium-sized enterprises. These so-called Mittelstand firms are often qualified as the “backbone” of the German economy. They are not only seen as the key to Germany’s quick postwar recovery, but also as a type of company allowing the German economy to cope with huge external shocks such as the recession in consequence of the worldwide financial crisis of 2008/2009. However, this claim was yet not backed by suitable empirical evidence. Because Mittelstand firms are often hard to identify in official statistics, the existing empirical evidence on Mittelstand firms in general is still comparatively scarce.

This paper contributes to broadening the empirical evidence on the role of Mittelstand firms by delivering an analysis of the relative performance of Mittelstand firms throughout the Great Recession of 2008/2009. Basically, it delivers supporting evidence for the stability hypothesis. After identifying Mittelstand firms as owner-managed SMEs based on objective, measurable features in the ifo Business Survey we find that Mittelstand firms in fact performed better than non-Mittelstand firms throughout the Great Recession of 2008/2009. This result proves to be robust in various stability tests. Interestingly enough, this finding does not carry over to the case where Mittelstand firms are classified in accordance to their subjective self-assessment, which often differs from the objective classification. Thus, we might conclude that further empirical studies of the role of Mittelstand firms should be based on objective criteria rather than on self-assessments of firms. As mentioned earlier, the empirical literature on the relative performance of Mittelstand firms is still in its infancy. This is primarily due to the fact that most available databases do not allow identifying Mittelstand firms properly as they typically lack information on ownership and management structure. Against this background it is highly desirable to increase efforts to build up databases which contain the necessary information as a basis for a more systematic empirical foundation of research on Mittelstand firms.^14^ It is often argued that Mittelstand firms are also more innovative and more engaged in vocational training, thereby delivering important inputs for the economy as a whole. While there is some macroeconomic evidence in favor of these arguments,^15^ micro-evidence on these issues is still missing. Moreover, almost all existing empirical evidence on Mittelstand firms currently comes from Germany; future research should aim to broaden the picture by considering data from other countries.

Our empirical results deliver a strong argument against industrial policies, primarily aiming at developing large national champions and to protect them against foreign competitors and takeovers. While such policies have little tradition in Germany, where the Mittelstand is very popular, in February 2019 the Federal Minister of Economics announced a paradigm shift towards such a strategy in his “National Industrial Strategy 2030”.^16^ As we have shown at the example of firm data from Germany, large and non-owner-managed firms tend to react more volatile in times of crises as Mittelstand firms. Thus, the current renaissance of industrial policies might contribute to less crisis resistance, even in the home country of Mittelstand firms.

## Appendix: A. Data set preparation

### A.1 Merging the ifo micro data

The raw ifo data are not immediately applicable for our purpose, so we were in need of some data set preparations beforehand. Our main goal is to generate a comprehensive data set representing the total German economy by merging the four industries together. In a nutshell, we executed the following steps:

1. **Assigning unique variable names:** We had to rename the industry-specific answers to a question by giving each variable a unique name. This ensures that, for example, the entries of the question on the business situation in the final data set contains the answers from all four industries.
2. **Defining the survey identifier:** We defined a unique identifier for each industry to ensure industry-specific analyses.
3. **Defining the industry identifier:** We assigned to each product category a unique identifier that is perfectly comparable to the 2-digit industrial level code. For example, we assigned the value “29” to a car producer in manufacturing, which is identical to the official Classification Code of Economic Activities of the German Federal Statistical Office.
4. **Collapsing the time dimension:** We defined a consecutive time identifier that reduces the year-month-combination to a simple running number.
5. **Defining the firm identifier:** We had to calculate a unique firm identifier which is a combination of the plant number and the industrial code. This was necessary in order to conduct an analysis on the firm rather than on the product level. The German Mittelstand is defined on the firm level, thus, we had to deal with multiple records. We elaborate more on this issue in the next section.
6. **Cleaning:** We had to delete duplicates due to wrong coding in the original sources.
7. **Merging:** We merged the four single data sets to one comprehensive source representing the German economy.

### A.2 Firm identification

Each ifo survey is conducted at the product level, but the Mittelstand is a criterion that defines the firm type. Thus, multiple firm records might bias our quotas and therefore our empirical results. For this purpose we need to aggregate the firm- and product-specific survey results so that our cross-section dimension represents the firm instead of the product level. We do so by following the approach proposed by Link (2020).

**Table 7:** Questions asked in manufacturing

#	Indicator	Description	Type	Frequency
1	Business Situation (BS)	**Question:** “We assess our current business situation as [...]” **Answers:** (+) good, (=) satisfactory, or (–) bad.	Standard	Monthly
2	Stock of Finished Products (SFP)	**Question:** “We assess our current stock of finished products as [...]” **Answers:** (+) too small, (=) sufficient, or (–) too large; (X) no stock-keeping.	Standard	Monthly
3a	Current Orders (CO)	**Question:** “We assess our stock of current orders as [...]” **Answers:** (+) relatively large, (=) sufficient, or (–) too small.	Standard	Monthly
3b	Foreign Orders (FO)	**Question:** “We assess our stock of current orders for the export as [...]” **Answers:** (+) relatively large, (=) sufficient, or (–) too small; (X) no export activities.	Standard	Monthly
4	Demand Development (DD)	**Question:** “Compared to the previous month, our demand situation has [...]” **Answers:** (+) improved, (=) remained unchanged, or (–) worsen.	Standard	Monthly
5	New Orders (NO)	**Question:** “Compared to the previous month, our stock of orders has [...]” **Answers:** (+) increased, (=) remained almost unchanged, or (–) decreased.	Standard	Monthly
6	Production Realization (PR)	**Question:** “Compared to the previous month, production has [...]” **Answers:** (+) increased, (=) remained almost unchanged, or (–) decreased; (X) no remarkable domestic production.	Standard	Monthly
7	Price Development (PRD)	**Question:** “Compared to the previous month, our prices were [...]” **Answers:** (+) raised, (=) unchanged, or (–) lowered.	Standard	Monthly
8	Employment Development (ED)	**Question:** “Compared to the previous month, the number of our employees has [...]” **Answers:** (+) increased, (=) remained almost unchanged, or (–) decreased.	Standard	Monthly
9	Production Expectations (PE)	**Question:** “In the next 3 months, our production will [...]” **Answers:** (+) increase, (=) stay the same, or (–) decrease; (X) no remarkable domestic production.	Standard	Monthly
10	Price Expectations (PRE)	**Question:** “In the next 3 months, our prices will [...]” **Answers:** (+) increase, (=) stay the same, or (–) decline.	Standard	Monthly
11	Export Expectations (EXE)	**Question:** “In the next 3 months, the extent of our export business will [...]” **Answers:** (+) grow, (=) stay the same, or (–) decrease; (X) no export activities.	Standard	Monthly
12	Employment Expectations (EE)	**Question:** “In the next 3 months, our number of employees will [...]” **Answers:** (+) increase, (=) stay the same, or (–) decrease.	Standard	Monthly
13	Business Expectations (BE)	**Question:** “In the next 6 months, our business situation will be [...]” **Answers:** (+) rather favorable, (=) rather stay the same, or (–) rather unfavorable.	Standard	Monthly
14	Expectations Forecast (EF)	**Question:** “Currently, to forecast our business expectations is [...]” **Answers:** (+) easy, (+) rather easy, (–) rather difficult, or (–) difficult.	Standard	Monthly
15	Order Range (OR)	**Question:** “Our current orders come up with a production time of [...]” **Answer:** *XX* months.	Special	Quarterly (1st month)
16	Capacity Utilization (CU)	**Question:** “The current utilization of our equipment (customary full use of the capacity = 100%) amounts to [...]” **Answers:** 30%, 40%, 50%, 60%, 70%, 75%, 80%, 85%, 90%, 95%, 100%, *XX*% (if above 100%).	Special	Quarterly (1st month)
17	Technical Capacity (TC)	**Question:** “Given our current stock of orders and expected new orders in the next 12 months, our technical capacity is [...]” **Answers:** (+) more than sufficient, (=) sufficient, or (–) not sufficient.	Special	Quarterly (1st month)
18	Production Obstruction (PO)	**Question:** “Our production activities are currently obstructed [...]” **Answers:** (+) yes, or (–) no. If yes, because of the following factors: insufficient orders, lack of employees, lack of specialists, financing bottleneck, lack of material, insufficient technical capacity, and other factors.	Special	Quarterly (1st month)
19a	Competitive Position Domestic (CPD)	**Question:** “In the last 3 months, our competitive position on the domestic market has [...]” **Answers:** (+) increased, (=) remained unchanged, or (–) decreased.	Special	Quarterly (1st month)
19b	Competitive Position inside EU (CPIEU)	**Question:** “In the last 3 months, our competitive position inside the European Union has [...]” **Answers:** (+) increased, (=) remained unchanged, or (–) decreased; (X) no export activities.	Special	Quarterly (1st month)
19c	Competitive Position outside EU (CPOEU)	**Question:** “In the last 3 months, our competitive position outside the European Union has [...]” **Answers:** (+) increased, (=) remained unchanged, or (–) decreased; (X) no export activities.	Special	Quarterly (1st month)
20a	Return on Sales Surplus (ROSS)	**Question:** “Our last year’s return on sales (in % of net turnover) was in case of a surplus [...]” **Answers:** up to 1%, above 1% to 2%, above 2% to 3%, above 3% to 4%, above 4%, in fact ca. *XX*%.	Special	Quarterly (2nd month)
20b	Return on Sales Deficit (ROSD)	**Question:** “Our last year’s return on sales (in % of net turnover) was in case of a deficit [...]” **Answers:** 0% to -1%, below -1% to -2%, below -2% to -3%, below -3% to -4%, below 4%, in fact ca. *XX*%.	Special	Quarterly (2nd month)
21a	Total Investment Development (TID)	**Question:** “Our last year’s total investment [...]” **Answers:** (+) increased, (=) remained unchanged, or (–) decreased.	Special	Quarterly (2nd month)
21b	Building Investment Development (BID)	**Question:** “Our last year’s building investment [...]” **Answers:** (+) increased, (=) remained unchanged, or (–) decreased.	Special	Quarterly (2nd month)
21c	Equipment Investment Development (EID)	**Question:** “Our last year’s equipment investment [...]” **Answers:** (+) increased, (=) remained unchanged, or (–) decreased.	Special	Quarterly (2nd month)
21d	Software Investment Development (SID)	**Question:** “Our last year’s software investment [...]” **Answers:** (+) increased, (=) remained unchanged, or (–) decreased.	Special	Quarterly (2nd month)
22a	Investment Indicator (II)	**Question:** “Our total investment in the current year will [...]” **Answers:** (+) increase, (=) remain unchanged, or (–) decrease.	Special	Quarterly (2nd month)
22b	Building Investment Expectations (BIE)	**Question:** “Our building investment in the current year will [...]” **Answers:** (+) increase, (=) remain unchanged, or (–) decrease.	Special	Quarterly (2nd month)
22c	Equipment Investment Expectations (EIE)	**Question:** “Our equipment investment in the current year will [...]” **Answers:** (+) increase, (=) remain unchanged, or (–) decrease.	Special	Quarterly (2nd month)
22d	Software Investment Expectations (SIE)	**Question:** “Our software investment in the current year will [...]” **Answers:** (+) increase, (=) remain unchanged, or (–) decrease.	Special	Quarterly (2nd month)
23	Overtime (OT)	**Question:** “We currently work overtime [...]” **Answers:** (+) yes, or (–) no. If yes, more than customary: (+) yes, or (–) no.	Special	Quarterly (3rd month)
24	Short-time Work (STW)	**Question:** “We currently apply short-time work [...]” **Answers:** (+) yes, or (–) no.	Special	Quarterly (3rd month)
25	Short-time Work Expectations (STWE)	**Question:** “In the next 3 months, we expect to apply short-time work [...]” **Answers:** (+) yes, or (–) no.	Special	Quarterly (3rd month)
26	Lending Activities (LA)	**Question:** “In the previous 3 months, we have been in lending negotiations with banks [...]” **Answers:** (+) yes, or (–) no. If yes, the banks behaved: (+) accommodating, (=) normal, or (–) restrictive. If no, because: (1) no credit demand, or (2) other reasons.	Special	Quarterly (3rd month)

*Source:* ifo Business Survey Manufacturing; own translations

**Table 8:** Questions asked in construction

#	Indicator	Description	Type	Frequency
1	Business Situation (BS)	**Question:** “We assess our current business situation as [...]” **Answers:** (+) good, (=) satisfactory, or (–) bad.	Standard	Monthly
2	Current Orders (CO)	**Question:** “We assess our stock of current orders as [...]” **Answers:** (+) relatively large, (=) sufficient, or (–) too small.	Standard	Monthly
3	Order Range (OR)	**Question:** “Our current orders come up with an average production time of [...]” **Answer:** *XX* months.	Standard	Monthly
4	Cost Coverage (CC)	**Question:** “Our construction prices cover [...]” **Answers:** (+) more than our production costs, (=) our production costs, or (–) less than our production costs.	Standard	Monthly
5	Production Obstruction (PO)	**Question:** “Our production activities are currently obstructed [...]” **Answers:** (+) yes, or (–) no. If yes, because of the following factors: insufficient orders, order cancellation, lack of employees, lack of specialists, financing bottleneck, lack of material, unfavorable weather conditions, and other factors.	Standard	Monthly
6	Construction Activity (CA)	**Question:** “Compared to the previous 3 months, our construction activity has [...]” **Answers:** (+) increased, (=) remained almost unchanged, or (–) decreased.	Standard	Monthly
7	Order Development (OD)	**Question:** “Compared to the previous month, our stock of construction orders [...]” **Answers:** (+) increased, (=) remained almost unchanged, or (–) decreased.	Standard	Monthly
8	Price Development (PRD)	**Question:** “Compared to the previous month, our construction prices were [...]” **Answers:** (+) raised, (=) unchanged, or (–) lowered.	Standard	Monthly
9	Construction Expectations (CE)	**Question:** “In the next 3 months, our construction activity will [...]” **Answers:** (+) increase, (=) stay the same, or (–) decrease.	Standard	Monthly
10	Price Expectations (PRE)	**Question:** “In the next 3 months, our construction prices will [...]” **Answers:** (+) increase, (=) stay the same, or (–) decline.	Standard	Monthly
11	Business Expectations (BE)	**Question:** “In the next 6 months, our business situation will be [...]” **Answers:** (+) rather favorable, (=) rather stay the same, or (–) rather unfavorable.	Standard	Monthly
12	Expectations Forecast (EF)	**Question:** “Currently, to forecast our business expectations is [...]” **Answers:** (+) easy, (+) rather easy, (–) rather difficult, or (–) difficult.	Standard	Monthly
13	Capacity Utilization (CU)	**Question:** “The current utilization of our machine capacity (customary full use of the capacity = 100%) amounts to [...]” **Answers:** 30%, 40%, 50%, 60%, 70%, 75%, 80%, 85%, 90%, 95%, 100%, *XX*% (if above 100%).	Standard	Monthly
14	Employment Expectations (EE)	**Question:** “In the next 3 months, our number of employees will [...]” **Answers:** (+) increase, (=) stay the same, or (–) decrease.	Standard	Monthly
15	Employment Development (ED)	**Question:** “Compared to the previous month, the number of our employees has [...]” **Answers:** (+) increased, (=) remained almost unchanged, or (–) decreased.	Standard	Monthly
16	Lending Activities (LA)	**Question:** “In the previous 3 months, we have been in lending negotiations with banks [...]” **Answers:** (+) yes, or (–) no. If yes, the banks behaved: (+) accommodating, (=) normal, or (–) restrictive. If no, because: (1) no credit demand, or (2) other reasons.	Special	Quarterly (3rd month)

*Source:* ifo Business Survey Construction; own translations

**Table 9:** Questions asked in retail trade

#	Indicator	Description	Type	Frequency
1	Business Situation (BS)	**Question:** “We assess our current business situation as [...]” **Answers:** (+) good, (=) satisfactory, or (–) bad.	Standard	Monthly
2	Stock of Finished Products (SFP)	**Question:** “We assess our current stock of finished products as [...]” **Answers:** (+) too small, (=) sufficient, or (–) too large; (X) no stock-keeping.	Standard	Monthly
3	Turnover Development (TOD)	**Question:** “Compared to the month of the previous year, our turnover have [...]” **Answers:** (+) increased, (=) remained unchanged, or (–) decreased.	Standard	Monthly
4	Price Development (PRD)	**Question:** “Compared to the previous month, our selling prices were [...]” **Answers:** (+) raised, (=) unchanged, or (–) lowered.	Standard	Monthly
5	Employment Development (ED)	**Question:** “Compared to the previous month, the number of our employees has [...]” **Answers:** (+) increased, (=) remained almost unchanged, or (–) decreased.	Standard	Monthly
6	Price Expectations (PRE)	**Question:** “In the next 3 months, our selling prices will [...]” **Answers:** (+) increase, (=) stay the same, or (–) decline.	Standard	Monthly
7	Order Expectations (OE)	**Question:** “In the next 3 months, our orders will [...]” **Answers:** (+) increase, (=) stay the same, or (–) decrease; (X) no remarkable domestic production.	Standard	Monthly
8	Employment Expectations (EE)	**Question:** “In the next 3 months, our number of employees will [...]” **Answers:** (+) increase, (=) stay the same, or (–) decrease.	Standard	Monthly
9	Business Expectations (BE)	**Question:** “In the next 6 months, our business situation will be [...]” **Answers:** (+) rather favorable, (=) rather stay the same, or (–) rather unfavorable.	Standard	Monthly
10	Expectations Forecast (EF)	**Question:** “Currently, to forecast our business expectations is [...]” **Answers:** (+) easy, (+) rather easy, (–) rather difficult, or (–) difficult.	Standard	Monthly
11	Turnover Obstruction (TOO)	**Question:** “Our turnover are currently obstructed [...]” **Answers:** (+) yes, or (–) no. If yes, because of the following factors: weak demand, lack of employees, lack of specialists, financing bottleneck, lack of real estate, insufficient office equipment, unfavorable weather conditions, and other factors.	Special	Quarterly (1st month)
12a	Local Footfall (LOFO)	**Question:** “In the previous quarter, the average footfall at our local position was [...]” **Answers:** (+) high, (=) seasonal, or (–) low; (X) no local position.	Special	Quarterly (1st month)
12b	Online Footfall (ONFO)	**Question:** “In the previous quarter, the average footfall at our online presence was [...]” **Answers:** (+) high, (=) seasonal, or (–) low; (X) no online presence.	Special	Quarterly (1st month)
13	Lending Activities (LA)	**Question:** “In the previous 3 months, we have been in lending negotiations with banks [...]” **Answers:** (+) yes, or (–) no. If yes, the banks behaved: (+) accommodating, (=) normal, or (–) restrictive. If no, because: (1) no credit demand, or (2) other reasons.	Special	Quarterly (3rd month)

*Source:* ifo Business Survey Retail Trade; own translations

**Table 10:** Questions asked in wholesale

#	Indicator	Description	Type	Frequency
1	Business Situation (BS)	**Question:** “We assess our current business situation as [...]” **Answers:** (+) good, (=) satisfactory, or (–) bad.	Standard	Monthly
2	Stock of Finished Products (SFP)	**Question:** “We assess our current stock of finished products as [...]” **Answers:** (+) too small, (=) sufficient, or (–) too large; (X) no stock-keeping.	Standard	Monthly
3	Turnover Development (TOD)	**Question:** “Compared to the month of the previous year, our turnover have [...]” **Answers:** (+) increased, (=) remained unchanged, or (–) decreased.	Standard	Monthly
4	Price Development (PRD)	**Question:** “Compared to the previous month, our selling prices were [...]” **Answers:** (+) raised, (=) unchanged, or (–) lowered.	Standard	Monthly
5	Employment Development (ED)	**Question:** “Compared to the previous month, the number of our employees has [...]” **Answers:** (+) increased, (=) remained almost unchanged, or (–) decreased.	Standard	Monthly
6	Price Expectations (PRE)	**Question:** “In the next 3 months, our selling prices will [...]” **Answers:** (+) increase, (=) stay the same, or (–) decline.	Standard	Monthly
7	Order Expectations (OE)	**Question:** “In the next 3 months, our orders will [...]” **Answers:** (+) increase, (=) stay the same, or (–) decrease; (X) no remarkable domestic production.	Standard	Monthly
8	Employment Expectations (EE)	**Question:** “In the next 3 months, our number of employees will [...]” **Answers:** (+) increase, (=) stay the same, or (–) decrease.	Standard	Monthly
9	Business Expectations (BE)	**Question:** “In the next 6 months, our business situation will be [...]” **Answers:** (+) rather favorable, (=) rather stay the same, or (–) rather unfavorable.	Standard	Monthly
10	Expectations Forecast (EF)	**Question:** “Currently, to forecast our business expectations is [...]” **Answers:** (+) easy, (+) rather easy, (–) rather difficult, or (–) difficult.	Standard	Monthly
11	Turnover Obstruction (TOO)	**Question:** “Our turnover are currently obstructed [...]” **Answers:** (+) yes, or (–) no. If yes, because of the following factors: weak demand, lack of employees, lack of specialists, financing bottleneck, lack of real estate, insufficient office equipment, unfavorable weather conditions, and other factors.	Special	Quarterly (1st month)
12	Lending Activities (LA)	**Question:** “In the previous 3 months, we have been in lending negotiations with banks [...]” **Answers:** (+) yes, or (–) no. If yes, the banks behaved: (+) accommodating, (=) normal, or (–) restrictive. If no, because: (1) no credit demand, or (2) other reasons.	Special	Quarterly (3rd month)

*Source:* ifo Business Survey Wholesale; own translations

**Table 11:** Questions asked in services

#	Indicator	Description	Type	Frequency
1	Business Situation (BS)	**Question:** “We assess our current business situation as [...]” **Answers:** (+) good, (=) satisfactory, or (–) bad.	Standard	Monthly
2	Current Orders (CO)	**Question:** “We assess our stock of current orders as [...]” **Answers:** (+) relatively large, (=) sufficient, or (–) too small.	Standard	Monthly
3	Employment Development (ED)	**Question:** “Compared to the previous month, the number of our employees has [...]” **Answers:** (+) increased, (=) remained almost unchanged, or (–) decreased.	Standard	Monthly
4	Price Development (PRD)	**Question:** “Compared to the previous month, our prices were [...]” **Answers:** (+) raised, (=) unchanged, or (–) lowered.	Standard	Monthly
5	Order Development (OD)	**Question:** “Compared to the previous month, our stock of orders [...]” **Answers:** (+) increased, (=) remained almost unchanged, or (–) decreased.	Standard	Monthly
6	Business Development (BD)	**Question:** “In the past 3 months, our business situation has [...]” **Answers:** (+) improved, (=) remained unchanged, or (–) worsen.	Standard	Monthly
7a	Turnover Development (TOD)	**Question:** “Compared to the previous 3 months, our turnover have [...]” **Answers:** (+) increased, (=) remained unchanged, or (–) decreased.	Standard	Monthly
7b	Turnover Development (TOD)	**Question:** “Compared to the previous month, our turnover have [...]” **Answers:** (+) increased, (=) remained unchanged, or (–) decreased.	Standard	Monthly
8	Turnover Expectations (TOE)	**Question:** “In the next 3 months, our turnover will [...]” **Answers:** (+) increase, (=) remain unchanged, or (–) decrease.	Standard	Monthly
9	Employment Expectations (EE)	**Question:** “In the next 3 months, our number of employees will [...]” **Answers:** (+) increase, (=) stay the same, or (–) decrease.	Standard	Monthly
10	Price Expectations (PRE)	**Question:** “In the next 3 months, our prices will [...]” **Answers:** (+) increase, (=) stay the same, or (–) decline.	Standard	Monthly
11	Business Expectations (BE)	**Question:** “In the next 6 months, our business situation will be [...]” **Answers:** (+) rather favorable, (=) rather stay the same, or (–) rather unfavorable.	Standard	Monthly
12	Expectations Forecast (EF)	**Question:** “Currently, to forecast our business expectations is [...]” **Answers:** (+) easy, (+) rather easy, (–) rather difficult, or (–) difficult.	Standard	Monthly
13	Business Obstruction (BO)	**Question:** “Our business is currently obstructed [...]” **Answers:** (+) yes, or (–) no. If yes, because of the following factors: weak demand, lack of employees, lack of specialists, financing bottleneck, lack of technical capacity, insufficient office equipment, unfavorable weather conditions, and other factors.	Special	Quarterly (1st month)
14	Demand Satisfaction (DS)	**Question:** “Is it currently possible for you to satisfy an increase in demand with the technical capacity at hand?” **Answers:** (+) yes, or (–) no. If yes, we can increase our business activity by *XX*%.	Special	Quarterly (1st month)
15	Lending Activities (LA)	**Question:** “In the previous 3 months, we have been in lending negotiations with banks [...]” **Answers:** (+) yes, or (–) no. If yes, the banks behaved: (+) accommodating, (=) normal, or (–) restrictive. If no, because: (1) no credit demand, or (2) other reasons.	Special	Quarterly (3rd month)

*Source:* ifo Business Survey Services; own translations

The firm aggregation is done within each ifo survey (e.g., manufacturing). If a car producer, for example, reports its business situation for three different products (e.g., cars, trucks, and motorcycles), we can densify these answers to calculate the business situation of the firm within manufacturing. However, the ifo data do not allow us to calculate firm-specific answers across the four surveys. If, for example, this car producer also has a product line or focus in services, we are not able to densify the answers from manufacturing and services as the ifo surveys do not contain cross-sectoral identifiers. Based on experiences and talks to employees of the ifo Institute, this is not a crucial issue as cross-sectoral reporting is negligible.

The aggregation within each survey is done as follows. First, we define a unique time identifier by collapsing the year and month dimension to one single running number. Second, we calculate a firm ID by grouping two ifo-specific identifiers together: the running number and the firm identification number. Whereas the running number is defined by a product group and assigned consecutively to new participants, the firm identification number is a combination of the sector to which the specific product is belonging to and an identifier for the firm. Especially the latter identifier is not public, but based on postal information of the firm. Third, we define the aggregation level by grouping the time identifier and the firm ID dimension. This leaves us with a unique identifier that connects the product-specific answers to the single firm within each survey. Finally, the product-specific answers to the survey questions (for example, the business situation) are averaged within the firm.

**Table 12:** Additional robustness results

	Dependent variable: business situation		
	Interaction model	Product level^‡^	Lagged Performance
Mittelstand Firms (*MS*)	− 0.27^∗∗∗^	− 0.14	− 0.12^∗∗∗^
	(0.10)	(0.11)	(0.05)
Crisis Period (*C**r**i**s**i**s*)	− 1.31^∗∗∗^	− 1.22^∗∗∗^	− 0.96^∗∗∗^
	(0.06)	(0.06)	(0.04)
Interaction Effect (*M**S* ∗ *C**r**i**s**i**s*)	0.43^∗∗∗^	0.48^∗∗∗^	0.36^∗∗∗^
	(0.08)	(0.09)	(0.05)
State-specific effects	YES	YES	YES
Sector-specific effects	YES	YES	YES
Interaction (*S**t**a**t**e* ∗ *S**e**c**t**o**r*)	YES	NO	NO
Lagged business situation	NO	NO	YES
Pseudo R-Squared	0.20	0.22	0.25
Observations	85,544	101,956	82,592

*Notes:* Ordered logit estimation with robust standard errors in parentheses; the inference for the product-level estimation is based on clustered standard errors instead (‡). ^∗∗∗^, ^∗∗^, and ^∗^ denote statistical significance to the 1%, 5%, and 10% level. The reference is the group of non-Mittelstand firms

According to our procedure, the number of multiple records within each survey is quite small. We observe 0.03% multiple records in manufacturing, 1.32% in trade, and 0.85% in services. The exception is construction with a multiple record count of 61.36%. This leaves us with a record of 14.19% for the total economy, and 0.63% by excluding the construction sector. Therefore, the bias introduced by multiple records might not be that large in manufacturing, trade, and services. However, it is a crucial issue to densify the answers to the firm level in construction.

### A.3 Balance sheet data

Due to the qualitative nature of the ifo Business Survey, only a few quantitative and firm-specific information are available. For our robustness analysis, we are therefore in need of further firm characteristics that might drive the firms’ resilience against economic downturns. One possible source of such information are balance sheet data, which can also be accessed at the EBDC. To be more concrete, the balance sheet data are provided by both the Amadeus- and the Hoppenstedt-Database. Both providers of balance sheet and income statement data publish a variety of firm characteristics and economic variables. As firm characteristics we can, for example, identify a firm’s founding year, its legal form, and whether it is listed on the stock exchange. The balance sheet information comprise, for example, the firms’ total equity, liabilities, and profits.

We merged both data sets together via a table provided by the EBDC that contains the identifiers needed for the merge.^17^ These identifiers are the firm ID, the month, the year, the industrial code, and a variable representing the federal state in which the respondent is located. Balance sheet data are only available on an annual basis, whereas the ifo survey results have a monthly frequency. We achieve the same time frequency by allocating the annual value of a balance sheet position to each month of that specific year. The merge, however, comes with the price that we lose a large number of observations as not all firms in the ifo Business Survey are liable to prepare a balance sheet.

## Footnotes

1 We discuss this definition in more depth in Section 2. See also Pahnke and Welter (2019) for a discussion of alternative definitions of Mittelstand firms.

2 For an overview on alternative databases on Mittelstand firms, see Schlömer-Laufen and Schneck (2020).

3 Firms which are managed by owners that did not found these firms (the normal case in more mature enterprises) are neglected in these studies.

4 Specifics of family firms versus owner-managed firms are discussed, for example, in Chrisman et al. (2004), Kets de Vries (1993), and Chu (2009).

5 The collection by Sauer and Wohlrabe (2020) gives a detailed introduction to the ifo Business Survey.

6 The survey data can be accessed via the LMU-ifo Economics & Business Data Center (EBDC). We employ the data sets for the four main industries: manufacturing (IBS-IND, 2016), construction (IBS-CON, 2016), trade (IBS-TRA, 2016), and services (IBS-SERV, 2016). Detailed descriptions of the data sets and the included variables can be found in various documentations available at the EBDC’s homepage: https://www.ifo.de/en/EBDC.

7 Appendix Appendix delivers a comprehensive overview of the monthly questions.

8 In our case, the Mittelstand dummy is time-varying because of the employment criterion. As we have only information on the owner structure in 2016, we assume that it does not change over time. However, this assumption should not drive our results as the ownership structure typically changes only rarely.

9 Another influence that might bias our results can be differences in the answering behavior across Mittelstand and non-Mittelstand firms. We checked whether both types of firms show any differences in their assessment of the current business situation for the period between 2011 and 2016 and find no such systematic differences across both groups.

10 Given the nature of the data and the applied empirical model, it is by no means trivial to report any kind of a “goodness-of-fit measure” such as a pseudo *R*^2^. We, nevertheless, make usage of the models’ pseudo log likelihoods and calculate a McFadden-style pseudo *R*^2^ that measures the improvement of the model including explanatory variables over the benchmark.

11 An indication in favor of the empirical relevance of this phenomenon is the fact that the average age of firms classifying themselves as Mittelstand firms is 48 years whereas those firms fulfilling the objective criteria is only 35 years.

12 We also tested whether firm size effects occur within the group of Mittelstand firms and find no systematic differences.

13 Due to computational reasons, we had to specify the model at a higher industrial level, namely for the four aggregates manufacturing, trade, construction, and services. This resulted in an ordered logit model with 60 additional variables (15 states times 4 industries).

14 For a discussion of this aspect see also Berlemann et al. (2018).

15 Macroeconomic evidence can be found in Berlemann and Jahn (2016) and Jahn (2018).

16 This strategy was criticized by German economists as well as business associations (see, e.g., Bofinger 2019, for a discussion).
